# Prior weight loss exacerbates the biological drive to gain weight after the loss of ovarian function

**DOI:** 10.14814/phy2.13272

**Published:** 2017-05-22

**Authors:** Vanessa D. Sherk, Matthew R. Jackman, Erin D. Giles, Janine A. Higgins, Rebecca M. Foright, David M. Presby, Ginger C. Johnson, Julie A. Houck, Jordan L. Houser, Robera Oljira, Paul S. MacLean

**Affiliations:** ^1^ Division of Endocrinology, Metabolism, and Diabetes Department of Medicine University of Colorado Anschutz Medical Campus Aurora Colorado; ^2^ Section of Endocrinology Department of Pediatrics University of Colorado Anschutz Medical Campus Aurora Colorado; ^3^ Department of Nutrition & Food Science Texas A&M University College Station Texas

**Keywords:** Energy balance, OVX, weight regain

## Abstract

Both the history of obesity and weight loss may change how menopause affects metabolic health. The purpose was to determine whether obesity and/or weight loss status alters energy balance (EB) and subsequent weight gain after the loss of ovarian function. Female lean and obese Wistar rats were randomized to 15% weight loss (WL) or ad libitum fed controls (CON). After the weight loss period, WL rats were kept in EB at the reduced weight for 8 weeks prior to ovariectomy (OVX). After OVX, all rats were allowed to eat ad libitum until weight plateaued. Energy intake (EI), spontaneous physical activity, and total energy expenditure (TEE) were measured with indirect calorimetry before OVX, immediately after OVX, and after weight plateau. Changes in energy intake (EI), TEE, and weight gain immediately after OVX were similar between lean and obese rats. However, obese rats gained more total weight and fat mass than lean rats over the full regain period. Post‐OVX, EI increased more (*P* ≤ 0.03) in WL rats (58.9 ± 3.5 kcal/d) than CON rats (8.5 ± 5.2 kcal/d), and EI partially normalized (change from preOVX: 20.5 ± 4.2 vs. 1.5 ± 4.9 kcal/day) by the end of the study. As a result, WL rats gained weight (week 1:44 ± 20 vs. 7 ± 25 g) more rapidly (mean = 44 ± 20 vs. 7 ± 25 g/week; *P* < 0.001) than CON. Prior obesity did not affect changes in EB or weight regain following OVX, whereas a history of weight loss prior to OVX augmented disruptions in EB after OVX, resulting in more rapid weight regain.

## Introduction

Menopause is a critical transition period that is associated with weight gain and an increased risk of obesity (Day et al. 2005; Duval et al. 2014; Melanson et al. 2015). Clinical studies suggest that, during this period, the weight gain in women is primarily brought about by decreased energy expenditure (Duval et al. 2014). While surgical ovariectomy (OVX) does not provide a perfect preclinical model of menopause, it does reflect some aspects of this transition with respect to the loss of ovarian estrogen production, a positive energy balance, and weight gain (Giles et al. 2010; Lovejoy et al. 2008; Witte et al. 2010). In this paper, we have merged this model with preclinical models of obesity and weight loss to examine how they influence the metabolic disturbances that occur with the loss of ovarian function.

Women are increasingly entering the menopausal transition with preexisting obesity and impairments in glucose and lipid metabolism (Catenacci et al. 2009; Flegal et al. 2016), but we do not have a clear picture of how excess weight and metabolic dysfunction influences the metabolic consequences of the loss of ovarian function. From one perspective, the accelerated weight gain on top of preexisting obesity may exacerbate the weight gain and metabolic problems that occur with the loss of ovarian function. Alternatively, it is plausible that obesity may protect from further weight gain during the menopausal transition because of the production of estrogens from adipose tissue and other peripheral tissues (Labrie 2015). Complicating our understanding of the interactions between obesity and menopause is the fact that premenopausal women frequently attempt to lose weight with variable rates of success. Reducing excess body weight can improve overall metabolic function (Magkos et al. 2016; McLaughlin et al. 2008; Rabol et al. 2009; Vitola et al. 2009), but it also induces a strong and persistent biological drive to regain weight (Jackman et al. 2006, 2010; Levin and Keesey 1998) that makes long‐term weight loss maintenance a difficult challenge (MacLean et al. 2015b). Given the high rates of obesity and weight loss efforts, it is important that we understand how obesity and weight reduction complicates the biological drivers of weight gain and metabolic disease at this critical transition.

Both the history of obesity and weight loss may independently change how loss of ovarian function affects metabolic health. Little is known about how a history of weight loss in formerly obese women affects metabolic changes associated with menopause. We hypothesize that the drive to gain weight during menopause will be even stronger in formerly obese women, compared to obese women who have not lost weight because weight loss and menopause‐induced changes in metabolism may be cumulative during that period (MacLean et al. 2010; Wallen et al. 2001).

The purpose of this study was to examine the impact of preexisting obesity, with or without weight loss, on the biological drive to gain weight after surgical OVX. We studied this interaction in rats that exhibited a polygenic predisposition to become obese or remain lean, when challenged with an obesogenic diet. First, we hypothesized that obesity would blunt OVX‐induced weight gain because of the preexisting metabolic perturbations in energy homeostasis and the production of estrogens in peripheral tissues. Second, we hypothesized that the biological drive to gain weight after OVX would be compounded with calorie‐restricted weight loss, but that this exacerbating effect in accelerating weight gain would be magnified in the obesity‐prone animals. Despite the limitations of these preclinical models in reflecting the human condition, their merger in this study provides valuable insights into how obesity, weight loss, and the loss of ovarian function interact to influence energy homeostasis.

## Methods

**Figure 1:**
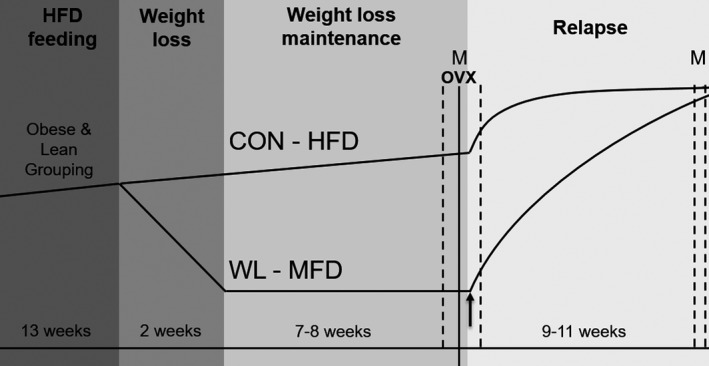
Study design and timeline. Dotted vertical lines and “M” designates the windows in which metabolic monitoring was performed. The black arrow designates the end of postOVX calorically limited (CL) feeding at the start of postOVX ad libitum (AL) feeding.

All procedures were approved by the University of Colorado Anschutz Medical Campus Institutional Animal Care and Use Committee. Five‐week‐old female Wistar rats were purchased from Charles River Laboratories (Wilmington, MA) and were individually housed on a 14:10 light–dark cycle and provided free access to water. An overview of the timeline and study design is shown in Figure 1. Briefly, rats were placed on a high‐fat diet (HFD; 46% kcal fat, Research Diets D12344, New Brunswick, NJ) at the time of arrival (~5 weeks of age). Using our established protocol of diet‐induced obesity (Giles et al. 2016), obesity‐prone (top tertile; obese *n* = 20) and obesity‐resistant (bottom tertile; lean *n* = 19) rats were identified 6 weeks later (~11 weeks of age) based on rate of weight gain and % body fat in response to the HFD. At 13 weeks of HFD (~17–18 weeks of age), rats were further split into control (CON) or weight loss (WL) groups, matching for body weight and body fat percentage.

After groups were split, CON rats remained on the HFD ad libitum as age‐matched untreated controls. WL rats were switched to an energy restricted provision of a medium‐fat diet (MFD; 25% kcal fat, Research Diets D07091301). We switched to a MFD for the WL rats based on the intent to better replicate the human condition, as weight loss in humans often includes some modification of fat intake. Each WL rat was provided a progression of calorie restriction from ~75% to ~55% of the calories eaten ad libitum to induce a 15% weight loss over 2 weeks. WL rats were then kept in energy balance at the reduced weight for 8 weeks by providing a limited portion of MFD just prior to the start of the dark cycle. Rats were ovariectomized (OVX) using a dorsal entry under isofluorane anesthesia (Park et al. 2010). To determine the effect of obesity on OVX‐induced changes in total and resting energy expenditure (TEE, REE) and spontaneous physical activity (SPA) prior to refeeding, WL rats were temporarily provided a calorically limited (postOVX‐CL) amount of MFD, whereas CON received ad libitum HFD during the 3–7 days of post‐OVX recovery and then for three additional days while they were in the metabolic monitoring system (described below). The postOVX‐CL provisions were based on anticipated calories needed to maintain energy balance, which was initially the same as their preOVX intake. Adjustments to the food allotment were made if a 2–3 days trend of weight loss or gain occurred. After 3 days of metabolic monitoring, animals were allowed to eat ad libitum on their respective diets for the remainder of the study. Rats were euthanized by exsanguination under anesthesia once their weight plateaued, which occurred 9–11 weeks postOVX.

### Body composition

Fat mass and fat free mass were measured using quantitative MR (qMR; EchoMRI, Houston, TX) at three time points: (1) before the initiation of weight loss, (2) prior to OVX, and (3) on the end of study.

### Metabolic monitoring

Energy intake (EI), SPA, TEE, and REE were measured in metabolic monitoring systems with indirect calorimetry (Oxymax CLAMS‐8M; Columbus Instruments, Columbus, OH). Each metabolic cage was equipped with an animal activity meter (Opto‐Max, Columbus Instruments, Columbus, OH), which allows for determination of total, ambulatory, and nonambulatory activity by monitoring the number of beam breaks within a one‐dimensional series of infrared beams. Activity was monitored continuously for 24 h. Metabolic monitoring was performed during the following conditions. Within 1 week of OVX, we performed daily vaginal lavages 4–5 h prior to the start of the dark cycle to determine the timing of the estrus phase within the estrous cycle to time the start of metabolic monitoring. If we detected the estrus phase via lavage, we skipped a day before performing a subsequent lavage. Metabolic monitoring began while rats were in the proestrus phase to allow the rats to go into estrus at a predictable time during monitoring. PreOVX monitoring lasted for 4 days. After recovery from OVX surgery (3–7 days), rats were monitored in the calorimeters for 3 days while on calorically limited (CL) provisions, plus 3 days of ad libitum (AL) refeeding. Finally, rats were monitored for 4 days during ad libitum feeding within a week of euthanasia (~9–11 weeks postOVX). The first day of preOVX and preSac monitoring were not included in analyses, as they were acclimation days. Metabolic rate (MR) was calculated from gas exchange measurements acquired every 14 min using the Weir (1949) equation: MR = 3.941 × VO_2_ + 1.106 × VCO_2_ − 2.17 × N, where N is urinary nitrogen. MR was averaged and extrapolated over 24 h to estimate TEE. Because of the ad libitum feeding patterns, REE was determined by taking the mean of the 3 lowest EE measurements during the light cycle. Energy balance (EB) was calculated as the difference between EI and TEE. Metabolic data were derived as mean values from 3 days in preOVX, 1–3 days in postOVX‐CL, 1–3 days in postOVX‐AL, and 1–3 days in preSac‐AL.

### Serum hormone and substrates

Blood was collected from the inferior vena cava at the time of euthanasia, and serum was stored at −80°C until analysis. All analyses were performed in duplicate. Plasma insulin and leptin were measured by ELISA (80‐INSRT‐E01 and 22‐LEPMS‐E01, respectively, ALPCO, Salem, NH). Limits of detection (LOD) were 0.124 ng/mL for insulin and 10 pg/mL for leptin. Colorimetric assays were used to measure plasma nonesterified fatty acids (NEFA; Wako Chemicals USA, Richmond, VA), glucose, triglycerides (TG), and total cholesterol (#TR15421, TR22321, and TR13521, respectively; Thermo Fisher Scientific, Waltham, MA).

### Statistical analyses

Data were analyzed using SAS version 9.4. This study was designed to test a difference in weight change and fat mass after refeeding (Maclean et al. 2011). Based on our previous work in males, a sample size of six was required to detect a 35 g difference in weight gain between CON and WL rats at 80% power and a *P*‐value of 0.05. Two‐way (phenotype, weight loss status) ANOVAs were used to test for baseline differences between CON and WL groups. Two‐way (phenotype, weight loss status) ANOVAs were then used to test the hypothesis that prior weight loss will augment the increases in weight and fat mass post‐OVX in lean and obese rats. The “Estimate” command within the Proc GLM (general linear model) was used for observing significant changes within group. We also performed similar two‐way ANOVAs on changes in EI, EE, EB, and SPA (secondary outcomes) as the mechanism for weight regain. Results in Table 2 include all data, but figures include paired data only. The level of significance was set at *P* < 0.05.

## Results

### Prior to the loss of ovarian function

**Figure 2:**
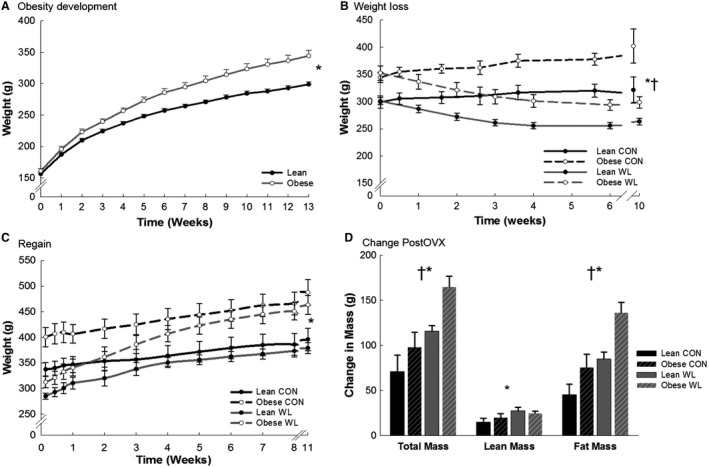
Obesity development (A) and weight loss before OVX (B), and weight regain after OVX (C) and change in mass after OVX (D). *N* = 19–20/group in (A). *N* = 8–11/group in (B–D). *P < 0.01, main effect of phenotype (obese > lean). ^†^ *P* < 0.01, main effect of weight loss (WL > CON).

**Figure 3:**
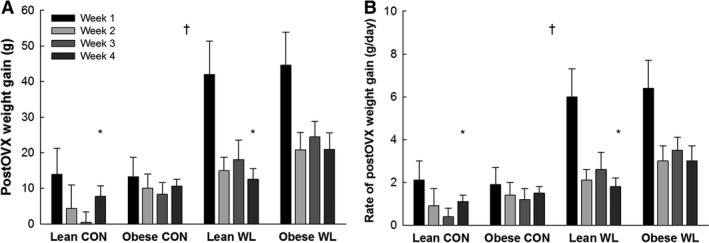
Rapid weight gain (A) and accelerated rate of gain (B) after the initiation of ad libitum feeding after OVX. *P = 0.028, main effect of phenotype (obese > lean) during week 4 only. ^†^ *P* ≤ 0.001, main effect of weight loss (WL>CON) at all time points.

Obese animals were heavier at the end of the 13‐week obesity development period than lean rats (Table 1, Pre Wt Loss; Fig. 2A). At the end of the weight reduction period (immediately prior to OVX), all groups had similar lean mass, and differences in body weight were driven by differences in fat mass (Table 1, Post Wt Loss; Fig. 2B). The effect of weight loss status on EB neared significance (*P* = 0.06), with EB being more positive in CON rats due to a ~50% higher EI and ~29% higher TEE (both *P* < 0.001; Table 2). Obese rats had higher TEE and REE (Table 2), but not EI (*P* = 0.08). Total and ambulatory activity in obese‐CON animals was approximately 25% lower than all other groups, but this was not significant (Fig. 3A).

**Table 1:** Total, lean, and fat mass preOVX and before weight loss, after weight loss just prior to OVX, and at the end of study. Only weight loss (WL) groups lost weight. Control (CON) groups continued ad libitum feeding

	CON		WL	
	Lean (*n* = 9)	Obese (*n* = 8)	Lean (*n* = 10)	Obese (*n* = 12)
Total mass (g)				
Pre wt loss^a^	295.2 ± 9.7	340.7 ± 9.7	300.7 ± 5.6	352.7 ± 12.7
Post wt loss^a^^,^ b	325.1 ± 15.8	389.9 ± 17.9	263.5 ± 6.2	298.7 ± 10.5
Final^a^	396.1 ± 21.9	488.0 ± 24.9	379.4 ± 10.6	463.6 ± 18.6
Lean mass (g)				
Pre wt loss	207.2 ± 7.8	211.7 ± 6.3	210.6 ± 4.3	219.1 ± 7.1
Post wt loss	221.4 ± 8.4	227.4 ± 17.9	205.2 ± 3.9	219.4 ± 6.5
Final	236.2 ± 10.9	247.4 ± 24.9	232.6 ± 4.8	243.9 ± 8.0
Fat mass (g)				
Pre wt loss^a^	60.6 ± 2.7	98.7 ± 6.8	61.9 ± 2.8	103.8 ± 7.6
Post wt loss^a^^,^ b	84.9 ± 7.8	130.8 ± 15.5	25.4 ± 2.7	47.6 ± 6.7
Final^a^	130.0 ± 13.9	206.6 ± 23.3	110.2 ± 9.3	184.1 ± 15.5

a *P* < 0.01 main effect of phenotype (obese > lean).

b *P* < 0.01 main effect of weight loss (WL > CON).

**Table 2:** Energy balance (EB), energy expenditure, energy intake before OVX, after OVX during caloric limitation (CL) and early ad libitum feeding (AL), and at end of study. Average of 3 days per feeding condition and time during preOVX due to cycle, 1–3 days postOVX; all data included

	CON		WL	
	Lean (*n* = 3–4)	Obese (*n* = 3–4)	Lean (*n* = 6–8)	Obese (*n* = 6–7)
EB				
PreOVX	2.8 ± 1.7	9.7 ± 7.3	−0.2 ± 1.5	0.2 ± 2.9
PostOVX CL	−8.2 ± 1.6	−0.2 ± 7.0	−1.1 ± 1.6	0.0 ± 5.1
PostOVX AL^b^	19.1 ± 1.2	30.5 ± 15.4	50.9 ± 2.2	48.4 ± 3.6
Final	3.9 ± 5.0	3.8 ± 2.3	5.7 ± 3.4	14.3 ± 2.9
TEE				
PreOVX^a^^,^ b	53.0 ± 0.7	56.9 ± 1.0	40.6 ± 1.7	45.6 ± 1.8
PostOVX CL^a^^,^ b	52.4 ± 1.3	58.1 ± 0.9	41.4 ± 2.1	45.7 ± 2.1
PostOVX AL	52.2 ± 2.1	57.4 ± 1.9	51.9 ± 2.1	52.7 ± 1.6
Final	53.9 ± 2.6	59.4 ± 4.8	53.4 ± 1.1	55.5 ± 2.2
REE				
PreOVX^a^^,^ b	36.1 ± 0.8	39.6 ± 0.7	28.4 ± 1.2	30.7 ± 0.9
PostOVX CL^b^	36.0 ± 1.4	41.0 ± 1.4	29.0 ± 1.7	31.3 ± 1.5
PostOVX AL	39.7 ± 0.9	40.7 ± 1.8	39.8 ± 1.7	39.3 ± 1.3
Final	37.6 ± 2.6	41.4 ± 4.3	37.1 ± 1.1	38.8 ± 1.7
Intake				
PreOVX^b^	55.8 ± 3.5	66.6 ± 7.7	40.4 ± 2.1	44.6 ± 10.9
PostOVX CL	44.2 ± 2.3	57.8 ± 6.1	40.4 ± 2.5	45.7 ± 6.0
PostOVX AL^b^	71.3 ± 3.2	88.0 ± 14.3	102.8 ± 3.4	101.1 ± 4.2
Final	57.8 ± 2.7	63.2 ± 3.3	59.1 ± 3.5	69.8 ± 4.7

EB, energy balance; TEE, total energy expenditure; REE, resting energy expenditure.

a *P* < 0.05 main effect of phenotype (Obese > Lean).

b *P* < 0.01 main effect of weight loss (WL ≠ CON).

### Effects of obesity on postOVX outcomes

**Figure 4:**
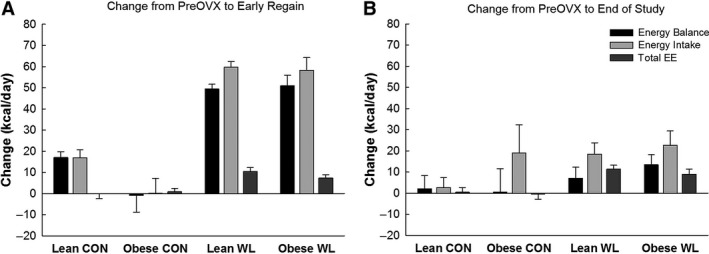
Changes in energy balance, energy intake, and total energy expenditure (TEE) from before OVX to the early refeeding phase (A) and to the end of the study (B). *P* < 0.05, main effect of weight loss for all variables for both time points.

**Figure 5:**
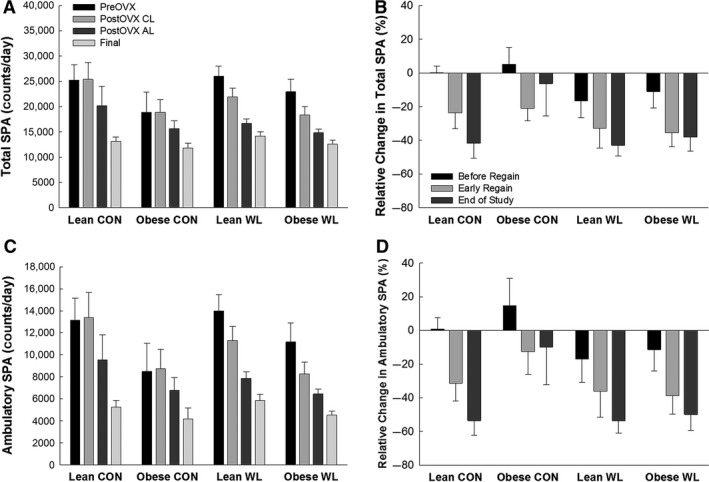
(A) Total spontaneous physical activity (SPA) at each time point. (B) Relative changes in total SPA from before OVX to the calorically limited phase (before regain) and early during the refeeding phase after OVX, and just prior to sacrifice. (C) Ambulatory spontaneous physical activity (SPA) at each time point. (D) Relative changes in ambulatory SPA from before OVX to the calorically limited (CL) phase (before regain) and early during the ad libitum (AL) refeeding phase after OVX, and just prior to sacrifice. No significant difference in change between groups.

The total amount of weight and fat gained during the entire postOVX period (Fig. 2C and D) was greater in obese rats when compared to lean (*P* < 0.01). There was no effect of obesity on the increase in EI and EE after OVX, and the resulting increase in positive EB (Table 2, Fig. 4A). Thus, because obese rats entered the postOVX phase with a higher positive EB, they gained more weight than lean animals over the postOVX period because EB remained more positive in obese rats. Obesity status also did not affect the decrease in physical activity (Fig. 5). Leptin was higher in obese rats (*P* < 0.01). There were no other significant differences in plasma measures.

### Effects of obesity and preOVX weight loss on postOVX outcomes

At week 5 postOVX, the rate of weight gain obese‐WL rats was higher than obese‐CON rats, but the rate of weight gain was similar between lean‐WL and lean‐CON (interaction: *P* = 0.038). There was a trend (*P* = 0.07) for phenotype × weight loss interaction for cholesterol levels, where a history of weight loss appeared to decrease cholesterol in obese rats only (Table 3).

**Table 3:** Plasma levels of hormones and metabolites at the end of the study

	CON		WL	
	Lean (*n* = 8)	Obese (*n* = 8)	Lean (*n* = 10)	Obese (*n* = 12)
Insulin (ng/mL)	1.16 ± 0.15	1.1 ± 0.2	0.95 ± 0.08	1.32 ± 0.16
Glucose (mg/dL)	152 ± 14	166 ± 18	163 ± 10	162 ± 15
Leptin (pg/mL)^a^	5453 ± 1017	8645 ± 2052	4527 ± 475	8918 ± 868
NEFA (mEq/L)	0.58 ± 0.06	0.56 ± 0.05	0.49 ± 0.07	0.62 ± 0.07
Trig (mg/dL)	60 ± 12	72 ± 9	59 ± 11	77 ± 10
Cholesterol (mg/dL)^a^	82 ± 5	107 ± 8	83 ± 5	88 ± 4

NEFA, nonesterifed fatty acids.

a *P* < 0.05, main effect of phenotype (obesity > lean).

### Effects of preOVX weight loss on postOVX outcomes

After OVX, weight gain in WL groups was higher (*P* ≤ 0.001) than CON groups through week 4 (Fig. 3). All WL rats regained and surpassed their preweight loss within 45 days of ad libitum feeding postOVX (mean ± SE = 14.5 ± 2.2 days). Lean‐WL rats regained 143.6 ± 1.8% of their lost weight and ended up 25.8 ± 1.9% heavier than their peak weight prior to weight loss. Obese‐WL rats regained 148.9 ± 3.9% of their lost weight, and ended up 24.7 ± 2.4% heavier than their weight prior to weight loss (at age 17–18 weeks). Meanwhile, lean‐CON was 34.3 ± 5.4% heavier than their weight just prior to OVX, and obese‐CON was 25.2 ± 2.4% heavier than their weight just prior to OVX. Thus, body weight of obese‐WL rats quickly surpassed those of lean‐CON and approached those of obese‐CON rats (Table 1, Fig. 2C). Weight (re)gain was predominately fat, with relatively small lean mass gains (Fig. 2D), in all groups. The total amount of weight, lean, and fat mass gained during the entire postOVX period (Fig. 2C and D) was greater in WL versus CON (*P* ≤ 0.02).

Rats with a history of prior weight loss demonstrated a greater increase in EI, TEE, and REE during the initial ad libitum feeding period post‐OVX (all *P* ≤ 0.002), and the increased EI, TEE, and REE persisted throughout the study (all *P* ≤ 0.03). There was a main effect of weight loss status for the initial (i.e., early refeeding phase) increase in EB (*P* < 0.01) and the change in EB from preOVX to the end of study (*P* = 0.04). Having a history of weight loss did not affect decreases in physical activity (all *P* > 0.14; Fig. 5).

## Discussion

The novel observation of this study is that weight loss greatly exacerbates the biological drive to gain weight after the loss of ovarian function. Contrary to our hypotheses, preexisting obesity did not affect the rate or amount of OVX‐induced weight gain with or without weight loss. In rats with prior weight loss, the substantial increase in food intake during the initial postOVX ad libitum feeding period (1–2 weeks) was the predominant contributor to a large, positive energy imbalance and resulting rapid weight regain. These observations suggest a greater impact of weight loss, rather than preexisting obesity status, in compounding the biological drive to gain weight after the loss of ovarian function.

We and others have shown the calorie‐restricted weight loss leads to a strong biological drive to regain weight (Higgins et al. 2011; Jackman et al. 2008; Levin and Keesey 1998; MacLean et al. 2006, 2009). Difficulties in maintaining weight loss include increased appetite and decreased satiety, which increases food intake, and decreased energy expenditure that is associated with decreases in body mass and thermic effect of food (Higgins et al. 2011; MacLean et al. 2006, 2015b). Our observations are consistent with this body of literature, but add to it by testing how this drive interacts with the loss of ovarian estrogen production. In this study, we combined these two paradigms and have shown for the first time that a history of weight loss prior to OVX more than doubled both positive energy imbalance and rate of weight gain after OVX compared to animals that had never lost weight. We have also observed that intact female relapsing rats have a smaller and less sustained early regain (R. M. Foright, V. D. Sherk, G. C. Johnson, M. R. Jackman, P. S. MacLean, unpubl. data). During regain, male rats will also exceed their preweight loss weight, but the time required to do so is influenced by the relative fat composition in the diet. When fed a HFD during refeed, regain took about 2 weeks, where regain took about 6 weeks with a low‐fat diet (Higgins et al. 2011; MacLean et al. 2006, 2009). Remarkably, the weight‐reduced rats ended up at a similar final body weight as control groups, which was markedly (~26% for lean, ~31% for obese) higher than their preweight loss weight. The implication is that although weight gain more readily plateaus in humans than in rats, formerly obese women entering the menopausal transition may be particularly vulnerable to weight gain during the transition. However, weight cycling does not appear to prevent postmenopausal women with obesity from achieving future weight loss (Mason et al. 2013).

It is established that the loss of estrogen induces a period of rapid weight gain (Santollo et al. 2010, 2011), and there is clinical evidence of a decrease in EE after the loss of ovarian function (Day et al. 2005; Melanson et al. 2015). As an additional level of control, our study design included a calorically limited phase after OVX to monitor changes in SPA and EE after OVX in the absence of overfeeding. Regardless of obesity or weight loss status, we did not detect a decrease in total or resting EE after OVX, and the metabolic flux while maintaining EB was very similar to preOVX. During the initial overfeeding, the initial increase in positive EB in these female rats was greater than that observed during relapse in male rats (MacLean et al. 2006, 2009). The expected decrease in SPA (Giles et al. 2010) was delayed until refeeding commenced, but that may have been due to increased appetite without an increase in food availability. SPA quickly decreased below preOVX levels once ad libitum access began, but did not create the expected decrease in TEE. The relapsing phase partly precludes measuring REE during a fasted condition. Therefore, the increases in TEE and REE, and weight gain, was attributed to an increase in the thermic effect of feeding (TEF) because of the increase in food consumption, as previously observed (Giles et al. 2010; McElroy and Wade 1987). An increased TEF may upwardly bias the REE measurements. Groups may have also had different temporal patterns of food intake, introducing variability into how much TEF could bias the REE measurements, and possibly SPA patterns. These factors should be considered when interpreting the energy expenditure data, as they make the independent effects of OVX and obesity on longer term changes in energy expenditure more difficult to determine.

The lack of decrease in total energy expenditure suggests that weight loss and OVX must be having a compounding effect on the drivers of food intake. Mechanisms underpinning the accelerated gain due to overeating are unknown, but we hypothesize that OVX and prior weight loss have an additive or a synergistic effect on both central and peripheral signaling of satiety, hunger, and nutrient depletion. Extensive reviews of the mechanisms of OVX‐induced weight gain (Asarian and Geary 2013; Gupte et al. 2015; McElroy and Wade 1987; Sainsbury and Zhang 2012) and biological drive to regain (Maclean et al. 2011; MacLean et al. 2015a) have been published. Briefly, one such mechanism is through the regulation of leptin signaling in the hypothalamus. Circulating leptin levels decrease with weight loss as fat stores become depleted. The loss of estrogen has been shown to attenuate the decrease in food intake after leptin delivery to the hypothalamus (Clegg et al. 2006; Marangon et al. 2014). Thus, combining weight loss with OVX could lead to a condition of both low leptin and low sensitivity to leptin in the hypothalamus, creating an enormous drive to eat. It would be best to evaluate this possibility during the weight reduced state, whereas we only have tissues from this study at the end of relapse. We currently have ongoing studies to address the role of leptin in postOVX weight gain.

Peripheral adaptations to OVX and caloric restriction are also likely contributing to the initial disruption of energy balance. Caloric restriction induces a decrease in circulating insulin with an increase in insulin sensitivity (Capel et al. 2009; Hayes et al. 2007; Kirk et al. 2009; Lien et al. 2009; McLaughlin et al. 2008). OVX decreases insulin secretion from the pancreas, reduces muscle insulin sensitivity, and increases insulin clearance and degradation in the liver (Park et al. 2016; Santos et al. 2016). Weight loss and the loss of estrogen can each cause bone loss, which could alter the insulin‐sensitizing effects of osteocalcin (Ferron et al. 2008; Shapses and Sukumar 2012). Some of the hypothesized changes from combining weight loss and OVX would include an increase in hepatic glucose production, combined with decreased glucose uptake into muscle and bone (Cavalcanti‐de‐Albuquerque et al. 1985). There would also be a suppression of fat oxidation in muscle and bone and increased shunting of calories into adipose tissue (Jackman et al. 2008). The overarching combination would be a decreased ability to expend calories, and an increased storage of nutrients in adipose tissue.

We acknowledge that keeping CON rats on HFD while WL rats were switched to a MFD should be considered when interpreting the results. Possible alternative approaches would have been to also include an age‐ and diet‐matched control group that were provided a MFD without weight loss, or to allow the WL group to regain on a HFD. Considering the influence of fat composition on weight regain in males (Higgins et al. 2011; MacLean et al. 2009), we would expect that MFD would somewhat reduce weight gain in CON rats. If WL rats had been allowed to refeed on HFD, we would expect the rate of regain to be faster than what we observed. Therefore, although we sought to improve the ability to translate to humans, either of these alternative approaches could have further magnified the effect of prior weight loss on the biological drive to gain weight after the loss of ovarian function.

As we attempt to translate our observations to the human condition, it is important to reiterate the limitations of the OVX model as a reflection of the menopausal transition. Menopause is a process and not an event. OVX results in an abrupt removal of estrogen, while perimenopause may last for years and inflict highly variable states of ovarian function that eventually ends with a decline in ovarian function. Results from the SWAN study suggest a faster decline in estrogen may be more likely to occur in nonobese women, possibly introducing a confounder that further complicates the translation to humans (Tepper et al. 2012). It is possible that utilizing a more gradual chemically induced approach or following the natural loss of ovarian function may have revealed effects that are more reflective of the human condition. We acknowledge that aging could influence weight gain after the initial effect of OVX or early relapse wanes. Even so, the OVX model allows us to temporally align study outcomes in a manner that is very difficult to replicate in humans or these alternative preclinical approaches. It should also be noted that the timelines of weight gain, OVX‐induced weight gain, weight regain, and aging are much compressed in our rodents models as compared to humans (weeks vs. years). The changes in energy balance and weight gain may be more easily detectible in the preclinical models than in humans.

## Perspectives and Significance

Menopause is an inevitable process that is often accompanied with obesity or a history of weight loss. This trifecta creates a complex setting by which excessive weight gain and metabolic dysfunction can occur. We combined models of diet‐induced obesity, weight loss and regain, and the loss of ovarian function to gain novel insight into the energetic challenges of menopause. The results of this study indicate that prior weight loss exacerbates the biological drive to gain weight that occurs with the loss of ovarian function. In contrast to our expectations, preexisting obesity had no detectable effect on weight gain after the loss of ovarian function. Taken together, these observations suggest that, regardless of the level of adiposity, the menopausal transition may present a considerable challenge for women who have lost weight and are maintain the weight reduced state. The enhancement of appetite with a decreased drive to be physically active implies that even greater motivational efforts would be required to both restrain eating and maintain physical activity levels to maintain energy balance, when controlling each is already difficult for many.

## Conflict of Interest

No conflicts of interest, financial or otherwise, are declared by the authors.
